# Reconstruction of the Largest Pedigree Network for Pear Cultivars and Evaluation of the Genetic Diversity of the USDA-ARS National *Pyrus* Collection

**DOI:** 10.1534/g3.120.401327

**Published:** 2020-07-16

**Authors:** Sara Montanari, Joseph Postman, Nahla V. Bassil, David B. Neale

**Affiliations:** * Department of Plant Sciences, University of California, Davis, CA; † USDA Agricultural Research Service, National Clonal Germplasm Repository, Corvallis, OR

**Keywords:** population structure, germplasm characterization, single nucleotide polymorphism markers, biodiversity conservation, pear breeding

## Abstract

The USDA-ARS National Clonal Germplasm Repository (NCGR) in Corvallis, Oregon, maintains one of the world’s largest and most diverse living *Pyrus* collection. A thorough genetic characterization of this germplasm will provide relevant information to optimize the conservation strategy of pear biodiversity, support the use of this germplasm in breeding, and increase our knowledge of *Pyrus* taxonomy, evolution, and domestication. In the last two decades simple sequence repeat (SSR) markers have been used at the NCGR for cultivar identification and small population structure analysis. However, the recent development of the Applied Biosystems Axiom Pear 70K Genotyping Array has allowed high-density single nucleotide polymorphism (SNP)-based genotyping of almost the entire collection. In this study, we have analyzed this rich dataset to discover new synonyms and mutants, identify putative labeling errors in the collection, reconstruct the largest pear cultivar pedigree and further elucidate the genetic diversity of *Pyrus*.

The USDA-ARS National Clonal Germplasm Repository (NCGR) in Corvallis, Oregon, maintains one of the world’s largest and most diverse collection of *Pyrus* (Postman 2008a). It includes 2,300 clonal pear accessions and 364 seed lots, encompassing 36 different species or interspecific hybrids from 55 countries (Postman 2008b). This collection represents, therefore, a useful tool for population and evolutionary genetic studies in *Pyrus*, as well as a valuable source of material for breeding purposes. An understanding of the genetic diversity of this collection will better support the use of this germplasm for the improvement of scion and rootstock pear cultivars (Volk *et al.* 2019). Additionally, since in the past germplasm to be preserved at the NCGR was selected based on morphological, geographical and passport data, there is a need to implement molecular marker screening to verify the trueness-to-type of the accessions maintained, as well as to eliminate redundancies. Finally, the use of molecular markers will aid in the elucidation of parentage for hundreds of cultivars and breeding material in the collection. Accurate pedigree information is not only essential for proper parental selection in breeding programs, but it also allows geneticists to infer trait heritability, understand genetic correlations among phenotypes of interest, and estimate breeding values (Kouassi *et al.* 2009; Cellon *et al.* 2018; Piaskowski *et al.* 2018). Such validated pedigree information also enables more powerful marker association studies (Bink *et al.* 2007; Kumar *et al.* 2013).

In the last two decades numerous studies implemented the use of simple sequence repeat (SSR) markers at the NCGR for cultivar identification and germplasm characterization (Bassil *et al.* 2005; Volk *et al.* 2006; Bassil and Postman 2010; Evans *et al.* 2015). However, single nucleotide polymorphism (SNP) markers are more abundant in the genomes (Rafalski 2002) and, when applied in large numbers, they have been shown to outperform SSRs for population structure and genetic relatedness studies (Lemopoulos *et al.* 2019). Thanks to many recent technological advances, it is today possible to carry out high-density SNP-based genotyping of a large number of samples at a low cost per data point. Therefore, while SSRs are still the markers of choice for routine fingerprinting analyses, the use of SNPs for germplasm characterization has recently increased (Hinze *et al.* 2017; Arab *et al.* 2019; Rufo *et al.* 2019; Xia *et al.* 2019). Additionally, public SNP arrays could be used as common marker sets in separate studies of different germplasm collections, thereby providing an opportunity to have comparable analysis at a global level. Such knowledge would be instrumental for the identification of gaps and for the optimization of the conservation strategy at genebank collections worldwide (Urrestarazu *et al.* 2015).

In the past decade there has been a flurry of studies on the genetic diversity, population structure and phylogeny of subsets of local *Pyrus* germplasm collections (Chevreau *et al.* 2020). However only recently did the application of next-generation sequencing technologies and high density SNP-based genotyping lead to important discoveries about the degree of diversity among *Pyrus* species and increase our understanding of the evolution and domestication of this genus (Kumar *et al.* 2017; Wu *et al.* 2018; Kim *et al.* 2019).

In this study, we used high-density genotypic data generated with the recently developed Applied Biosystems Axiom Pear 70 K Genotyping Array (Montanari *et al.* 2019) to begin the characterization of the *Pyrus* collection held at the NCGR. By genotyping almost 2,000 samples, we discovered new synonyms and mutants, identified putative labeling errors at the collection, reconstructed the largest pedigree of pear cultivars, and further elucidated the genetic diversity of *Pyrus*.

## Material and Methods

### Plant material and genotyping

A total of 1,890 diploid *Pyrus* spp. samples, two haploids, and five intergeneric hybrids (×*Pyronia* = *Pyrus* × *Cydonia*; ×*Sorbopyrus* = *Sorbus* × *Pyrus*), were used for the analysis performed in this work (Table S1). Specifically, this list of 1,897 samples consisted of: i) 288 (including biological and technical replicates) that were screened with the draft Axiom Pear 700 K Genotyping Array by Montanari *et al.* (2019) (hereafter called Screening Panel); ii) 1,415 (including biological and technical replicates and the two haploids, two ×*Pyronia* and three ×*Sorbopyrus* accessions) genotyped with the Axiom Pear 70 K Genotyping Array by Montanari *et al.* (2019) (hereafter called Genotyping 1 Panel); iii) 194 additional accessions screened in this work with the Axiom Pear 70 K Genotyping Array (hereafter called Genotyping 2 Panel). From the Screening Panel, only the 275 samples that had passed genotyping standards were kept (Montanari *et al.* 2019), and only the genotypic information for the 71,363 SNPs that were also included in the 70 K array were used for the following analysis. For all other samples, the raw data were merged and re-analyzed, using a QC CR threshold of 96.385 (for details see Affymetrix Axiom Genotyping Solution – Data Analysis Guide, https://assets.thermofisher.com/TFS-Assets/LSG/manuals/axiom_genotyping_solution_analysis_guide.pdf).

The 71,363 SNPs of the 70 K array were aligned to the new Double Haploid (DH) Bartlett Genome (Linsmith *et al.* 2019) using BLAST (Altschul *et al.* 1990) as explained in Montanari *et al.* (2019), except with an identity threshold of 90%.

### Identification of duplicated samples

Among the SNPs that had high quality and unique alignments to the new pear genome, only the *PolyHighResolution* (PHR, according to the Affymetrix default parameters for diploid samples) were used for pairwise comparison of all 1,897 samples. Identity by state (IBS) values were computed using plink v1.90 (options--allow-extra-chr--distance square0 ibs). The available biological and technical replicates were used to set the IBS threshold for the identification of the duplicated samples. For each group of duplicates, the genotype with the lowest number of missing data were selected for subsequent analysis.

### Pedigree reconstruction

Five F_1_ crossing populations from the Washington State University (Guzman 2018) and the USDA-ARS Appalachian Fruit Research Laboratory (Zurn *et al.* 2020) pear breeding programs were used to identify erroneous SNPs based on Mendelian inheritance and then aid in the pedigree reconstruction of the *Pyrus* accessions. These populations consisted of: 63 offspring derived from ‘Bartlett’ × ‘Anjou’, 82 from ‘Bartlett’ × ‘Doyenne du Comice’, 97 from ‘Old Home’ × ‘Bartlett’, 83 from ‘Potomac’ × ‘El Dorado’, and 85 from NJA2R59T69 × ‘Bartlett’, for a total of 410 trios. They were added to 260 known trios/duos from the sample set of this study. SNPs were filtered for missing data (removed if >2%), and then a Mendelian test was run on the 670 known trios using *trio.check* as described in Montanari *et al.* (2019), and SNPs with an error rate > 5% were removed. This marker dataset was used to compute the relationship inference between each pair of samples with the KING-robust method (Manichaikul *et al.* 2010), implemented in the R package SNPRelate v1.14.0 (Zheng *et al.* 2012). As demonstrated by Manichaikul *et al.* (2010), Linkage Disequilibrium (LD) pruning is not necessary for application of this method. The computed kinship coefficients (*k*), which represents the probability that two random alleles from the two samples are identical by descent, can be used to identify first-degree relationships. Within those, the value of IBD0, *i.e.*, the probability that the two samples share zero alleles identical by descent, can be used to distinguish parent-offspring (PO) from full-sib (FS) relationships. The theoretical value of *k* in first-degree relationships is 0.25, and the IBD0 is 0 in PO and 0.25 in FS. However, in practice such values deviate from the theoretical ones and depend on the characteristics of the specific population under study. The values of *k* and IBD0 for 90% of the pairwise combinations in the known trios, confirmed by Mendelian test (< 10% error rate), were used to set the thresholds to apply in this study. All new trios and duos that were identified upon applying the set thresholds for *k* and IBD0 were again tested for Mendelian errors, and those with error rate < 1.5% were considered true. A second search of PO and FS was carried out by refining the inference criteria (in this case using *k* and IBD0 values for 95% of the newly confirmed relationships), and were again confirmed by Mendelian test (<1.5% error rate). New PO and FS relationships were compared with the literature (Hedrick *et al.* 1921; Jacob 1998; Mielke and Smith 2002; Simard and Michelesi 2002; Pasqualini *et al.* 2006; Sawamura *et al.* 2008; Bassil and Postman 2010; Postman *et al.* 2013; Bell *et al.* 2014; Morgan 2015) and the information stored at the NCGR website (https://www.ars.usda.gov/ARSUserFiles/20721500/catalogs/pyrcult.html) regarding year and country of origin and believed parentage, when available. Pedigree networks were designed with the R package network v1.13 (Butts 2008) and with the software Helium (Shaw *et al.* 2014).

### Population structure analysis

The SNP dataset was pruned for LD using an r^2^ threshold of 0.80 in plink v1.90 (options--allow-extra-chr--indep-pairwise 50 5 0.80), but not filtered for MAF, as the Axiom 70K SNPs were carefully chosen to include rare alleles that would correctly depict population structure (Montanari *et al.* 2019). A Principal Component Analysis (PCA) was run using the R package SNPRelate, and the graph for the first two PCs plotted with ggplot2 and using a species-based color-coding for the samples. The software fastSTRCTURE (Raj *et al.* 2014) was then run to infer the population structure, using a hierarchical approach. First, inferences were performed for K = 2 to 30, with 15 replicates per K, and then both the fastSTRUCTURE algorithm for multiple choices of K and the Evanno’s ad hoc procedure (Evanno *et al.* 2005) were performed in an attempt to choose the optimal number of subpopulations. Because of the complexity of the structure, another round of structure inference was run separately on the subpopulations and the admixed group identified at K = 2; up to K = 22 was used in this second round. Finally, Clumpp (Jakobsson and Rosenberg 2007) was used to summarize data from the 15 replicates and obtain mean Q-values. Samples with Q ≥ 0.75 were assigned to the relative subpopulation, and plots were designed with the program Structure Plot v2.0 (Ramasamy *et al.* 2014). Additionally, a PCA was run again for each of the subpopulations and the admixed identified at K = 2 in the initial structure analysis. Results from the PCA and the structure analysis were compared, and used to identify samples that had been likely assigned to the wrong species and propose a new classification. PC1 *vs.* PC2 plots for each subpopulation used a color-coding based on the new proposed classification.

In an attempt to further resolve the complexity of one of the subpopulations identified (the Occidental group), a discriminant analysis of principal components (DAPC) was also carried out (Jombart *et al.* 2010). DAPC was performed using the R package adegenet v2.1.2 (Jombart 2008; Jombart and Ahmed 2011). The optimal number of clusters was chosen running the *find.clusters* function for up to 90 clusters, and then examining the values of Bayesian Information Criterion (BIC) for each number of clusters. The function *dapc* was then run on the groups inferred with *find.clusters* at the chosen number of clusters and using the first 500 PCs and four discriminant functions. Results were plotted with ggplot2.

### Data availability

Supplemental data (Tables S1-S4; Figure S1; Files S1-S5) are provided through figshare. The genotyping data for the 1,749 samples that passed genotyping standards and 64,571 SNPs that had unique, high-quality alignment to the DH Bartlett Genome and that were classified as PHR are provided through the Genome Database for Rosaceae (GDR, https://www.rosaceae.org/, accession number tfGDR1042). Supplemental material available at figshare: https://doi.org/10.25387/g3.12186105.

## Results

### SNP genotyping and BLAST on the new ‘Bartlett’ genome assembly

A total of 1,474 samples from Genotyping1 and 2 Panels passed genotyping standards, which, together with the passed samples from the Screening Panel, summed up to 1,749 samples.

All 71,363 SNPs aligned to the DH Bartlett Genome, as expected. However, 965 SNPs were discarded after quality filtering of the alignments. Additionally, 4,638 SNPs aligned to multiple locations, and therefore were eliminated. Of the remaining 65,760 SNPs, 64,571 were classified as PHR and used for the subsequent analysis.

### Identification of mutants, synonyms and labeling errors, and pedigree reconstruction

In this study, a large number of replicates (77) was used as controls among the different plates and genotyping panels. The IBS threshold above which two samples were considered identical was set to 97.7%. A total of 1,113 genotypes were unique (*i.e.*, did not have any duplicate). Excluding the 77 replicated samples, 218 groups of identical genotypes were found, encompassing a total of 534 samples. Most of the groups included just two samples, but some others had 10 or more. The group with the largest number of identical genotypes included the duplicates of ‘Bartlett’ (a.k.a. ‘Williams’ Bon Chretien’) and consisted of 31 samples. Table S2 reports all the samples with identical genotypes found in this study, with notes about whether they were already known (as reported in Hedrick *et al.* (1921); Morgan (2015) and from NCGR passport data available through the GRIN-Global website), if they were biological or technical replicates, or if they are suspected to be sampling or labeling errors, based on the following results from pedigree reconstruction and structure analysis.

**Figure 1:**
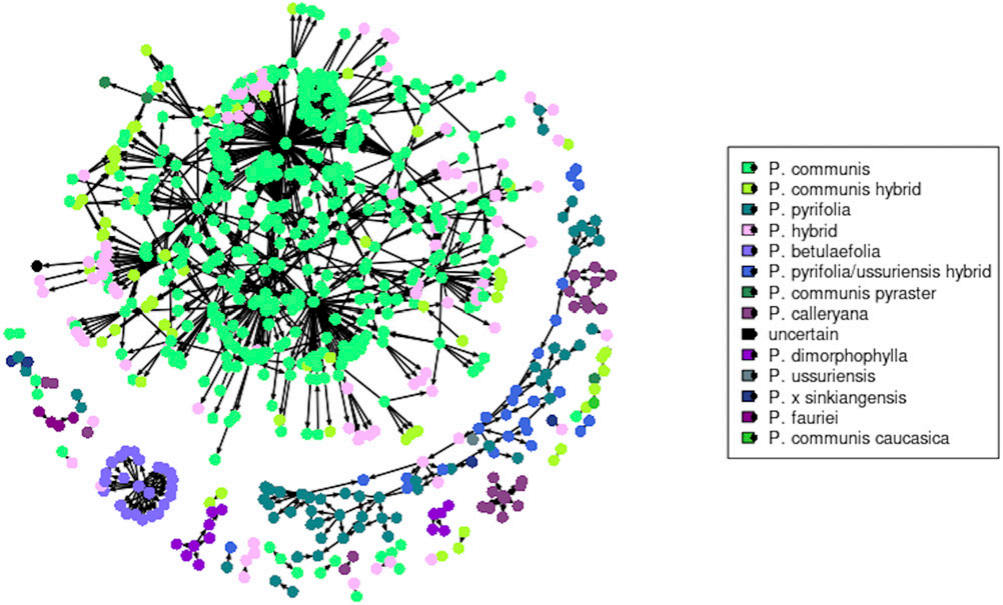
Pedigree network for all the trios and duos identified in this study. Each dot represents an accession and a color-coding based on the species is used, as shown in the legend on the right-hand side. Relationships are shown with an arrow from the parent to the offspring accession.

Removal of duplicates resulted in a number of 1,331 unique genotypes. After filtering for missing data and Mendelian error, 62,673 SNPs were left and 13 trios with > 10% error rate were eliminated, leaving 657 trios. In the first search, pairs of samples were assigned first-degree relationship if they had values of *k* ≥ 0.133, and among those PO were identified when IBD0 ≤ 0.005. In the second search, the thresholds for *k* and IBD0 were refined to 0.136 and 0.002, respectively. PO relationships were found for 723 accessions, across 13 species or interspecific hybrids (Figure 1); only 90 founders were identified (Table 1). In total, 139 trios/duos that were known before this study were hereby confirmed (at a more stringent threshold of 1.5% Mendelian error rate), and 498 new ones were identified. These numbers refer only to the *Pyrus* accessions evaluated in this study and do not include the five F_1_ crossing populations. Full information about discovered parentages can be found in Table S3, with relevant literature citations.

**Table 1:** List of the 90 founders from the inferred pedigree

Accession ID	Sample name	Taxon	Plant name
PI 542023	CPYR_1177.001	*Pyrus ×bretschneideri*	Tsu Li
PI 665781	CPYR_2638.002	*Pyrus ×bretschneideri*	Tsu Li 1
PI 542022	CPYR_1617.002	*Pyrus ×bretschneideri*	Xiangshui Li [Hsiang Sui-Li]
Q 27647	CPYR_2681.002	*Pyrus ×bretschneideri*	Xuehuali (Snowflake)
PI 506362	CPYR_1678.001	*Pyrus ×bretschneideri*	Ya Li
PI 665771	CPYR_2989.003	*Pyrus ×sinkiangensis*	Chinese Fragrant Pear
PI 540943	CPYR_653.001	*Pyrus betulaefolia*	OPR-110 P. betulifolia No. 1
PI 540946	CPYR_656.001	*Pyrus betulaefolia*	OPR-114 P. betulifolia No. 5
PI 540973	CPYR_1263.001	*Pyrus betulaefolia*	P. betulifolia OSU-3
PI 541108	CPYR_2189.001	*Pyrus calleryana*	Aristocrat (P. calleryana)
PI 617646	CPYR_2577.001	*Pyrus calleryana*	Bradford (P. calleryana)
PI 541083	CPYR_1601.001	*Pyrus calleryana*	P. calleryana OSU-10
PI 541053	CPYR_1264.003	*Pyrus calleryana*	P. calleryana OSU-2
PI 541018	CPYR_673.001	*Pyrus calleryana*	P. calleryana PC-5
PI 617505	CPYR_674.001	*Pyrus calleryana*	P. calleryana PC-6
PI 324124	CPYR_12.002	*Pyrus communis*	Akca
PI 264694	CPYR_23.002	*Pyrus communis*	Arganche
PI 654945	CPYR_2757.001	*Pyrus communis*	Bellissime d’Hiver
PI 541128	CPYR_52.002	*Pyrus communis*	Bergamote d’Ete
PI 541127	CPYR_51.002	*Pyrus communis*	Bergamotte d’Automne
PI 541523	CPYR_2131.001	*Pyrus communis*	Bergamotte de Baillargues
PI 260153	CPYR_53.001	*Pyrus communis*	Bergamotte Esperen
PI 541130	CPYR_56.002	*Pyrus communis*	Besi d’Hery
PI 654936	CPYR_2706.001	*Pyrus communis*	Bessemianka
PI 295083	CPYR_64.003	*Pyrus communis*	Beurré d’Arenberg
PI 541145	CPYR_78.002	*Pyrus communis*	Beurré Gris
PI 307539	CPYR_83.002	*Pyrus communis*	Beurré Inflancka
PI 541148	CPYR_86.002	*Pyrus communis*	Beurré Millet
PI 617587	CPYR_2510.002	*Pyrus communis*	Blanquilla (=Spadona)
PI 541387	CPYR_1165.001	*Pyrus communis*	Bosc - OP-5
PI 541305	CPYR_103.001	*Pyrus communis*	Brandy
PI 541163	CPYR_139.004	*Pyrus communis*	Citron de Carmes (Madeleine)
PI 654920	CPYR_2449.001	*Pyrus communis*	Colmar d’Ete
PI 541168	CPYR_156.001	*Pyrus communis*	Conference
PI 541183	CPYR_202.003	*Pyrus communis*	Early Harvest (=Chambers)
PI 392319	CPYR_205.002	*Pyrus communis*	Ecmianka
PI 231889	CPYR_230.001	*Pyrus communis*	Fondante de Charneu
PI 541191	CPYR_233.001	*Pyrus communis*	Forelle
PI 264194	CPYR_244.004	*Pyrus communis*	Gieser Wildeman
PI 260161	CPYR_490.004	*Pyrus communis*	King Sobieski
CPYR 2992	CPYR_2992.001	*Pyrus communis*	Kings Valley Pear 1
PI 541215	CPYR_346.001	*Pyrus communis*	Lemon
PI 130990	CPYR_1113.001	*Pyrus communis*	Madame Verte
Q 24302	CPYR_2978.001	*Pyrus communis*	Malti
PI 541233	CPYR_393.003	*Pyrus communis*	Messire Jean
PI 255616	CPYR_410.001	*Pyrus communis*	Napoleon
PI 541456	CPYR_431.001	*Pyrus communis*	Old Home
PI 541242	CPYR_451.002	*Pyrus communis*	Petit Blanquet
PI 541245	CPYR_466.002	*Pyrus communis*	President Loubet
PI 541256	CPYR_496.001	*Pyrus communis*	Rousselet de Reims
PI 541444	CPYR_1516.002	*Pyrus communis*	Stuttgarter-Geishirtle (= Zuckerbirne)
PI 260162	CPYR_578.001	*Pyrus communis*	Tonkowietka
PI 541281	CPYR_602.004	*Pyrus communis*	White Doyenne
PI 541282	CPYR_603.002	*Pyrus communis*	White Star
PI 638016	CPYR_2826.001	*Pyrus communis*	Yaquina (Payson)
PI 665773	CPYR_2859.001	*Pyrus communis*	Zutica
PI 337437	CPYR_687.001	*Pyrus communis* subsp. *caucasica*	P. communis ssp. caucasica - Stavropol
PI 483401	CPYR_1551.002	*Pyrus communis* subsp. *pyraster*	Crna Poloska
PI 325930	CPYR_1390.001	*Pyrus dimorphophylla*	P. dimorphophylla - Japan
PI 617507	CPYR_776.001	*Pyrus fauriei*	P. fauriei MSU5768
PI 260200	CPYR_1275.001	*Pyrus* hybrid	Cherry Pear
PI 541711	CPYR_239.002	*Pyrus* hybrid	Garber
PI 483372	CPYR_1526.002	*Pyrus* hybrid	Ilinka
PI 312503	CPYR_2386.001	*Pyrus* hybrid	Michurin Beurré Zimnaya (Winter)
PI 541239	CPYR_433.002	*Pyrus* hybrid	Orel No. 15
PI 617526	CPYR_1494.001	*Pyrus* hybrid	P. betulifolia 2 x P. call. 2
PI 541768	CPYR_1239.001	*Pyrus* hybrid	P. pashia x P. calleryana
PI 541776	CPYR_1315.001	*Pyrus* hybrid	P. ussuriensis x P. calleryana
PI 541812	CPYR_1702.001	*Pyrus* hybrid	South Dakota E-31
PI 134606	CPYR_573.002	*Pyrus* hybrid	Tioma
PI 541859	CPYR_725.002	*Pyrus nivalis*	P. nivalis P-91 (pure)
PI 228012	CPYR_178.002	*Pyrus pyrifolia*	Doitsu
PI 541897	CPYR_270.001	*Pyrus pyrifolia*	Hawaii
PI 352641	CPYR_294.001	*Pyrus pyrifolia*	Imamura Aki
PI 228013	CPYR_296.002	*Pyrus pyrifolia*	Ishiiwase
PI 541898	CPYR_303.003	*Pyrus pyrifolia*	Japanese Golden Russet
PI 97348	CPYR_1119.001	*Pyrus pyrifolia*	Meigetsu
PI 654923	CPYR_2642.002	*Pyrus pyrifolia*	Nepal 5053
PI 224196	CPYR_413.001	*Pyrus pyrifolia*	Nijisseiki
PI 392318	CPYR_428.001	*Pyrus pyrifolia*	Okusankichi
PI 541927	CPYR_1018.001	*Pyrus pyrifolia*	P. pyrifolia from A. Donovan house
PI 278731	CPYR_533.001	*Pyrus pyrifolia*	Sivaganga Estate
CPYR 2892	CPYR_2892.002	*Pyrus sachokiana*	P. sachokiana GE-2006-114
PI 541985	CPYR_27.002	*Pyrus ussuriensis*	Ba Li Xiang [Ba Li Hsiang]
PI 617537	CPYR_2338.001	*Pyrus ussuriensis*	Chien Li
PI 315064	CPYR_268.001	*Pyrus ussuriensis*	Hang Pa Li
PI 541990	CPYR_288.002	*Pyrus ussuriensis*	Huangxianshui Li [Huang Hsing Sui Li]
PI 541993	CPYR_291.001	*Pyrus ussuriensis*	Hung Li
PI 267863	CPYR_455.002	*Pyrus ussuriensis*	Ping Guo Li [Pingo Li]
PI 542007	CPYR_1157.002	*Pyrus ussuriensis*	Tzu Ma Li

A small number of accessions appeared to be the main founders in *P. communis*, and they include ancient and commercially important cultivars. For example, ‘White Doyenne’, believed to be the ancient cultivar ‘Doyenné Blanc’ originated in 1652, and possibly the same as the earlier ‘Pera Ghiacciuola’ described in 1559 (Hedrick *et al.* 1921), is the parent of 56 accessions, which are themselves involved in four more pedigree generations. ‘White Doyenne’ offspring of note are ‘Duchesse d’Angouleme’, ‘Bartlett’, ‘Comtesse de Paris’, ‘Anjou’ and ‘Coscia’. ‘Duchesse d’Angouleme’, first reported in 1808 (Hedrick *et al.* 1921), is itself the parent of 30 cultivars, including ‘Doyenne du Comice’ (second parent inferred is ‘Glou Morceau’), ‘Roi Charles de Wurttemberg’ and ‘Beurré Clairgeau’, this last one also parent of 33 accessions. ‘Roi Charles de Wurttemberg’ (presumed origin 1886) appeared to be a backcross of ‘Beurré Clairgeau’ × ‘Duchesse d’Angouleme’ (Figure S1a). ‘Bartlett’, found in Berkshire (UK) in 1770 (Hedrick *et al.* 1921), is the parent of the largest number of accessions (156), as expected, including ‘Clapp Favorite’ and ‘Kieffer’. ‘Clapp Favorite’, whose parentage ‘Flemish Beauty’ (syn. ‘Lesnaya Krasavitza’) × ‘Bartlett’ was confirmed, is the founder for 17 accessions, eight of which appeared to have a hybrid ancestry between *P. communis* and subsp. *pyraster* or *caucasica* (Figure S1b). ‘Kieffer’, a US hybrid cultivar which was reported to have first fruited in 1863, is itself the parent of 15 accessions (Figure S1c). One of ‘Kieffer’’s offspring is BP-2, a rootstock selection that originated in 1928 in South Africa. A number of accessions indistinguishable from ‘Kieffer’ were identified at the repository, including ‘Burford Pear’, ‘Campas No. 2’, and ‘Hermit’. Also, five accessions that were collected together in Pakistan (namely Nak I, Khan Tangoo I, India IC 20821, Kharnak I and Kharnak II) had the same genotype as ‘Kieffer’ (Table S2). The old Belgian cultivar ‘Comtesse de Paris’ had the same genotype as ‘Flemish Beauty’ (here its known synonym ‘Lesnaya Krasavitza’ was used). The Romanian cultivar ‘Rosii Untoase’, ‘Parker’ (claimed to be selected in Minnesota) and ‘Southworth’ also turned out to be identical to ‘Comtesse de Paris’. ‘Southworth’ was reported to be a synonym of ‘Vermont Beauty’ (Morgan 2015), which was not confirmed here. However, ‘Southworth’ and ‘Parker’ were donated to the NCGR by the same nursery, thus ‘Southworth’ might be a labeling error. ‘Comtesse de Paris’ is the founder to 23 accessions, including *P. communis* and hybrids. ‘Anjou’, whose first record was in the UK in the early XIX Century, turned out to have originated from ‘White Doyenne’ × ‘Sucre Verte’, this last one being an old cultivar known since 1670 and an inferred offspring of ‘Bergamotte d’Automne’ (Figure S1a). ‘Anjou’ is itself the parent of 30 accessions. It appeared that the labels for ‘Coscia’ and ‘Coscia Tardive’ had been swapped at the repository. ‘Coscia’ was reported to have originated in the late XVII Century, while ‘Coscia Tardive’ is known only since 1910 (Morgan 2015); they turned out to be connected by a PO relationship. Taking into account the swapped identity, ‘Coscia’ was inferred as offspring of ‘Blanquilla’ (syn. ‘Spadona’) × ‘White Doyenne’, and ‘Coscia Tardive’ originated from ‘Coscia’ × ‘Beurré Giffard’, this last one also a descendent of ‘White Doyenne’. Additionally, a number of cultivars known to be offspring of ‘Coscia’ were confirmed, in particular ‘Coscia Precoce’, ‘Butirra Precoce Morettini’ (‘Coscia’ × ‘Bartlett’), ‘Santa Maria’ (‘Coscia’ × ‘Bartlett’), ‘Etrusca’ (‘Coscia’ × ‘Ilinka’), ‘Butirra Rosata Morettini’ (‘Coscia’ × ‘Beurré Clairgeau’), ‘Tosca’ (‘Coscia’ × ‘Bartlett’) and ‘Leopardo Morettini’ (‘Coscia’ × ‘Beurré Easter’) (Figure S1d). These provided further evidence to support the swapped identity of ‘Coscia’ and ‘Coscia Tardive’ at the repository.

The old cultivars ‘Beurré Gris’ and ‘Glou Morceau’ showed a PO relationship, however it is unclear which one originated first. ‘Beurré Gris’ (syn. ‘Beurré Brown’) might be as early as 1628 or could have originated in 1867 in France (Morgan 2015), while ‘Glou Morceau’ was released in 1759 and introduced to France in 1806 (Hedrick *et al.* 1921; Morgan 2015). ‘Glou Morceau’ is the parent of 43 accessions, which gave rise to two more generations of cultivars. ‘Rousselet de Reims’ (inferred as a synonym of ‘Petite Rousselet’) is the founder of a five generation-pedigree. This cultivar is centuries old, it may even date back to the Roman age (Hedrick *et al.* 1921). The old cultivar ‘Verte Longue d’Automne’, first mentioned in 1628, appeared to be an offspring of ‘Rousselet de Reims’ and ‘Bergamotte d’Automne’. This last one was first reported in 1536 and is the parent of ten cultivars and the founder of a four generation-pedigree. ‘Seckel’, found in the USA in the mid XVIII Century, was inferred as an offspring of ‘Rousselet de Reims’ and ‘White Doyenne’, and is itself a parent of 19 accessions. ‘Winter Nelis’, a Belgian cultivar from the early XIX Century, turned out to be an offspring of ‘Besi de La Motte’ (first reported in 1685), which originated from ‘Bergamotte d’Automne’. ‘Winter Nelis’ is the parent of 17 accessions.

‘Old Home’ was confirmed as the parent of the erroneously named rootstock series ‘Old Home × Farmingdale’ (OH×F), as well as of ‘Pyrodwarf’ (‘Old Home’ × ‘Conference’), OH 20 and OH 50 (‘Old Home’ × ‘Bartlett’), BU 2/33 –Pyro II (‘Old Home’ × ‘Glou Morceau’), OH 11 – Pyriam, and QR 708-2, QR 708-12 and QR 708-36 (BP-2 × ‘Old Home’). The pollen parent of the OH×F rootstocks was again confirmed to be ‘Bartlett’, as already reported by Postman *et al.* (2013), except for OH×F 247 and 512 that resulted from a cross between ‘Old Home’ × ‘Anjou’.

In *P. pyrifolia*, a high degree of inbreeding from the cultivar ‘Nijisseiki’ was observed, as previously reported (Nishio *et al.* 2016) (Figure S1e). Furthermore, several accessions here identified as hybrids between *P. pyrifolia* and *P. ussuriensis* are related to each other, with the cultivar ‘Hau Kai’ having a central role in their pedigree (Figure S1f). ‘Hau Kai’ is a very old cultivar from Liaoning (Northeast) China that turned out to be an offspring of ‘Tzu Ma Li’ × ‘Ba Li Shian’. ‘Man Yuan Xiang’, also an old cultivar from Northeast China, resulted a synonym of ‘Hau Kai’ (Table S2). Finally, two accessions were inferred to be the founders of all the *P. betulaefolia* held at the NCGR: *P. betulaefolia* OSU-3 (CPYR 1263.001) and OPR-114 *P. betulaefolia* No. 5 (identical to OPR-111 *P. betulaefolia* No. 2), parents of 15 and 19 accessions, respectively. Both of these accessions are seedling selections from seeds collected in China and brought to Oregon and implemented in the pear rootstock breeding program there. Accession CPYR 1255.001 was given the same name of *P. betulaefolia* OSU-3, however its genotype was identical to that of *P. betulaefolia* OPR-260 and it was inferred to be an offspring of CPYR 1263.001 (Table S2, Table S3).

Uncertainties remain for cultivars of commercial or breeding importance. For example, the pedigree of ‘Bosc’, one of the main cultivars in the US Pacific Coast, was not resolved, and doubts persist about the identity of ‘Louise Bonne d’Avranches’ (a.k.a. ‘Louise Bonne Jersey’) at the NCGR. Two accessions of ‘Louise Bonne d’Avranches’ and its *panachee* mutant were analyzed, and they all turned out to be different from each other. Accessions CPYR 2106.001 and CPYR 2106.002 are likely to be either sampling errors at the time of leaf collection, or mis-labeling at the repository, while the accession of the mutant *panachee* (CPYR 2491.001) was inferred to be parent of the cultivar ‘Princess’, which was indeed thought to be a seedling of ‘Louise Bonne d’Avranches’. However, it also appeared to be identical to ‘Marie Louise’. The identity of ‘Marie Louise’ is also uncertain, since it was confirmed as parent of ‘Laxton’s Early Market’, but not of ‘Marie Louise d’Uccle’ (Table S2).

### Population structure

**Figure 2:**
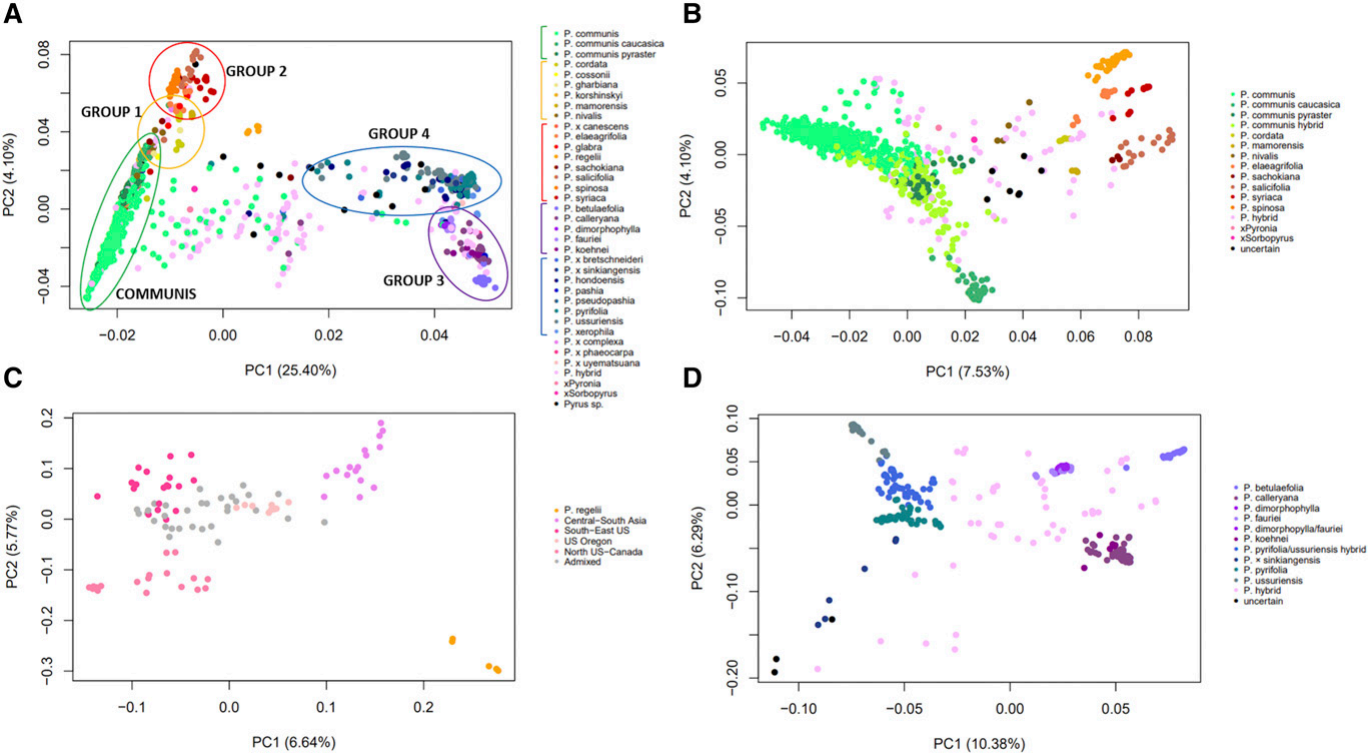
Principal component analysis plots. PC1 *vs.* PC2 plots are shown for a) all accessions; b) Occidental accessions; c) Admixed accessions; and d) Oriental accessions. Colors are assigned based on the known species assignment for a, and on the new species assignment proposed in this study for b, c and d. The percentages of variation accounted for by each PC1 and PC2 are displayed on the axes. In plot a the major groups of species are shown with circles on the chart, and with bars on the legend.

A total of 60,866 SNPs passed LD pruning and were used to examine the population structure and its consistency with the geographic-based grouping of the *Pyrus* species as reported in Challice and Westwood (1973) and in Montanari *et al.* (2019) (Table 2). The first four PCs explained, respectively, 25.40, 4.10, 3.87 and 2.42% of the overall genetic diversity (Table S4). The PC1 *vs.* PC2 plot (total of 29.5% of explained diversity; Figure 2a) depicted the two major groups of Occidental and Oriental pears, with a number of interspecific hybrids in between. Additionally, the three slightly overlapping clusters of the groups *P. communis* (including *P. communis*, *P. communis* subsp. *caucasica* and *P. communis* subsp. *pyraster*), Group 1 (species that are considered wild relatives of *P. communis*) and Group 2 (Middle East/Central Asia arid-adapted species) could be identified within the Occidental cluster, and the two groups Group 3 (East Asian “pea” pear species) and Group 4 (East Asian large-fruited cultivars and wild relatives) were distinguishable within the Oriental cluster.

**Table 2:** Classification of Pyrus species into different groups as reported in Montanari *et al.* (2019)

Occidental species	Oriental species
Group Communis	Group 3 (East Asian “pea” pears)
*Pyrus communis*	*Pyrus betulaefolia*
*Pyrus communis* subsp. *caucasica*	*Pyrus calleryana*
*Pyrus communis* subsp. *pyraster*	*Pyrus calleryana* f. *graciliflora*
Group 1 (Europe, North Africa – *P. communis* wild relatives)	*Pyrus dimorphophylla*
*Pyrus cordata*	*Pyrus fauriei*
*Pyrus cossonii*	*Pyrus koehnei*
*Pyrus gharbiana*	Group 4 (East Asian large-fruited cultivars and wild relatives)
*Pyrus korshinskyi*	*Pyrus ×bretschneideri*
*Pyrus mamorensis*	*Pyrus ×sinkiangensis*
*Pyrus nivalis*	*Pyrus hondoensis*
Group 2 (Middle East/Central Asia arid-adapted species)	*Pyrus pashia*
*Pyrus ×canescens*	*Pyrus pseudopashia*
*Pyrus elaeagrifolia*	*Pyrus pyrifolia*
*Pyrus glabra*	*Pyrus ussuriensis*
*Pyrus regelii*	*Pyrus xerophila*
*Pyrus sachokiana*	
*Pyrus salicifolia*	
*Pyrus spinosa*	
*Pyrus syriaca*	

**Figure 3:**
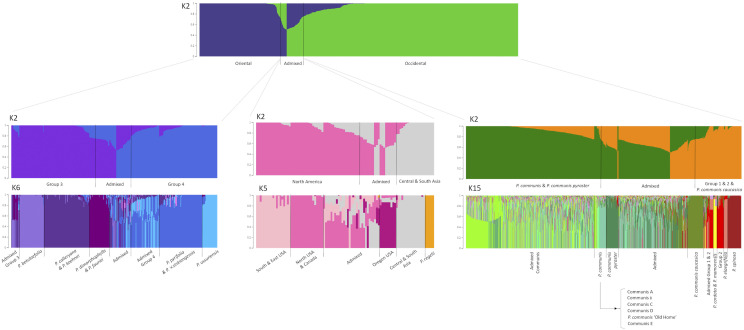
Hierarchical population structure analysis plots. The first plot shows the structure of all samples at K = 2, and the plots below show the structure of the Oriental, the Admixed and the Occidental groups at K = 2 and at the respective optimal Ks.

It was difficult to identify the optimal number of subpopulations on the overall population, therefore a hierarchical approach was applied. At K = 2, the two major groups of Occidental and Oriental pears were clearly identified, and the analysis was repeated for each of these subpopulations, as well as for the admixed group (Figure 3). Optimal values of K for the Occidental population were 12 to 15, according to the Evanno’s procedure (the fastSTRUCTURE algorithm for multiple choices of K gave uncertain results). At K = 2, one subpopulation included the pure *P. communis* and the *P. communis* subsp. *pyraster* samples, and the other one included *P. communis* subsp. *caucasica* and Group 1 and Group 2 accessions; a large number of admixed samples was found. At K = 15, the following subpopulations were identified: *P. cordata* with *P. mamorensis*; *P. elaeagrifolia*; *P. spinosa*; the rest of the Group 2 species (*P. salicifolia*, *P. syriaca* and *P. sachokiana*); *P. communis* subsp. *caucasica*; *P. communis* subsp. p*yraster*; six separate groups of pure *P. communis*; Group 1/Group2 hybrids; some more complex hybrids; and a large number of samples with admixture of different *P. communis* groups and subspecies. Within the Oriental population, at K = 2 the two subgroups Group 3 and Group 4 could be separated, and at the optimal number of K = 6 the following subpopulations were identified: *P. betulaefolia*; *P. calleryana* with *P. koehnei*; *P. dimorphophylla* with *P. fauriei*; *P. ussuriensis*; and *P. pyrifolia* with *P*. ×*sinkiangensis*. Similar to the Occidental group, samples with admixture of Group 3 species, samples with admixture of Group 4 species, and more complex hybrids admixed from the two groups were also found. Some subpopulations, apparently based on geographical origin, could be identified even among the Occidental/Oriental admixed. At K = 2, a group of North American and a group of Central and South Asian hybrids could be distinguished. At the optimal value of K = 5 a subpopulation for *P. regelii* could be separated from the Central and South Asian samples; a group of Northern USA and Canada hybrids and a group of South-Eastern USA hybrids could be identified within the North American subpopulation; and a group of accessions developed in Oregon, USA could be separated from the other admixed.

**Figure 4:**
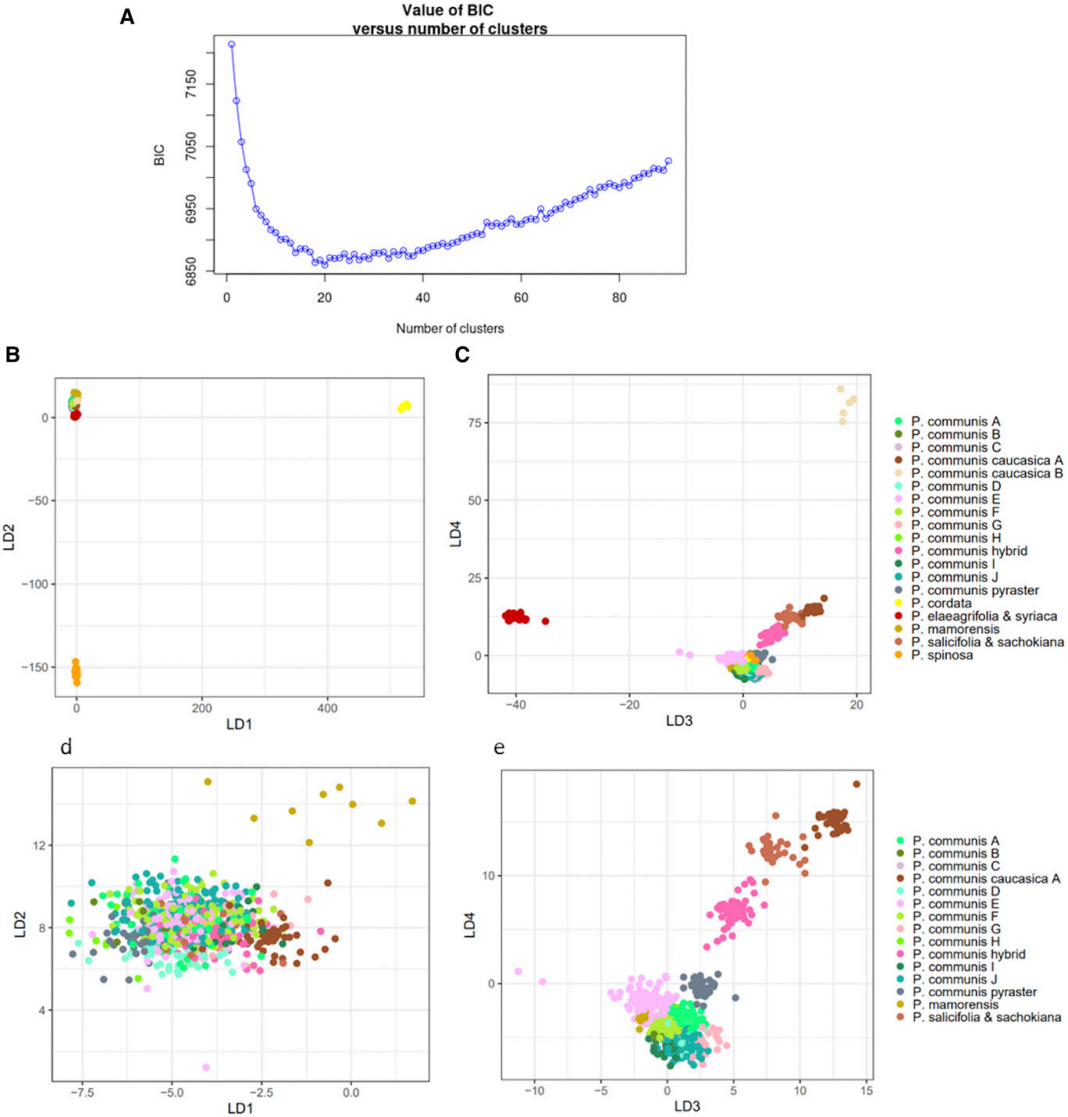
Discriminant analysis of principal components (DAPC) in the Occidental group. In a) the plot of BIC values *vs.* number of clusters; in b) the discriminant functions 1 *vs.* 2 (LD1 *vs.* LD2) plot and in c) the LD3 *vs.* LD4 plot for all groups identified with the DAPC; in d) the LD1 *vs.* LD2 plot and in e) the LD3 *vs.* LD4 plots for all groups identified with the DAPC excluding *P. cordata*, *P. spinosa*, *P communis caucasica* B and *P. elaeagrifolia* & *syriaca*.

A DAPC was also run on the complex Occidental group, using 20 clusters, which was the number of clusters with the lowest BIC value (Figure 4a). Ten separate groups of *P. communis* were identified (P. communis A through J), two of *P. communis* subsp. *caucasica* (P. communis caucasica A and B) and then one each for *P. communis* hybrid; *P. communis* subsp. *pyraster*; *P. cordata*; *P. elaeagrifolia* with *P. syriaca*; *P. mamorensis*; *P. salicifolia* with *P. sachokiana*; and *P. spinosa*. The plot of discriminant functions 1 and 2 (LD1 *vs.* LD2) showed that *P. cordata* and *P. spinosa* were the most diverse groups (Figure 4b), while the LD3 *vs.* LD4 plot showed that the groups *P. communis* subsp. *caucasica* B and *P. elaeagrifolia* & *syriaca* were the most diverse (Figure 4c). Results were then plotted again after removal of these four groups, and while at the LD1 *vs.* LD2 plot the groups were indistinguishable, except for *P. mamorensis* (Figure 4d), at the LD3 *vs.* LD4 plot clusters for each group were more compact, although still largely overlapping, except for *P. communis* subsp. *caucasica* A, *P. salicifolia* & *sachokiana*, and *P. communis* hybrid.

### Proposed new sample classification

Based on the results of the hierarchical structure and PC analysis, a new taxonomic classification was proposed for a number of accessions (Table S4). There were several accessions that resulted from hybridization between pure *P. communis* and its subspecies *caucasica* and *pyraster*, making their re-classification complicated. Several *P. nivalis* accessions appeared as mis-classified *P. communis* subsp. *caucasica* or *P. communis* hybrids. *P. korshinskyi* accessions appeared to be either *P. communis* subsp. *caucasica* or complex hybrids with various degrees of subsp. *caucasica* ancestry. Several accessions appeared mis-classified within Group 4. Here, two main subpopulations were identified, one for *P. ussuriensis* and one for *P. pyrifolia*; however, a number of samples that were assigned to *P. ussuriensis*, and a few assigned to *P. pyrifolia*, appeared to be hybrids between the two species. Additionally, approximately half of the *P*. ×*bretschneideri* and the *P. hondoensis* samples are likely hybrids between *P. ussuriensis* and *P. pyrifolia*, while the second half were reassigned to either one of the two species. Classification of *P*. ×*sinkiangensis* was rather difficult in this study. Of the eight samples analyzed, three were in the Occidental groups based on both analyses, and were reassigned to *P. communis* or *P. communis* subsp. *pyraster*; one was admixed between Oriental and Occidental; and four (of which two were PO related) formed a subpopulation with *P. pyrifolia*. The hierarchal structure analysis and PCA also allowed the inclusion in this species of two more samples that were mis-classified, bringing the number of putative *P*. ×*sinkiangensis* accessions to six. The small number of *P. pashia*, *P. pseudopashia* and *P. xerophila* samples appeared mis-classified.

## Discussion

The high-density genotyping performed in this study gave relevant information for germplasm conservation and *Pyrus* taxonomic classification, and it enabled a large pedigree reconstruction for cultivars held at the NCGR. The Axiom Pear 70 K Genotyping Array (Montanari *et al.* 2019) was a highly useful and efficient tool for high-throughput genotyping in a diverse number of *Pyrus* species. To the best of our knowledge, this is the largest germplasm characterization study performed in pear, encompassing 1,331 unique genotypes across 36 species, interspecific and intergeneric hybrids, and one of the largest pedigree reconstruction efforts in perennial fruit species, being on the same scale of the recent work in apple by Muranty *et al.* (2020).

### Genotyping tools are useful for optimization of conservation strategies at germplasm repositories

A large number of accessions that were collected in the wild or received from other germplasm repositories or donors from all over the world turned out to be identical to cultivars or accessions already present at the NCGR. All biological samples must undergo an expensive and time-consuming quarantine, pathogen testing and clean-up process before being released to the NCGR. Many of these efforts could have been avoided if synonymy or duplication with accessions already present at the NCGR collection was first determined by genotype comparison. Furthermore, potential labeling errors at the NCGR were flagged, and numerous previously unknown synonyms were discovered. Molecular markers could therefore provide a very useful tool to optimize material exchange between countries and avoid unnecessary expenses.

### Faulty historical pedigrees and high degree of inbreeding

The number of errors in historical pedigree records of pear cultivars appeared to be very high, with approximately 80 trios and duos that showed inconsistencies compared to that reported in the literature or in passport data at the NCGR. This was somehow expected, as several cultivars analyzed in this study are ancient and documentation is vague. However, this also indicates the necessity of using molecular markers to confirm or elucidate the parentage of new cultivars to be released, as well as of accessions recurrently used in breeding programs. It is, however, important to underline that the method applied in this study for the pedigree reconstruction could be subject to a certain degree of error and, even if a stringent threshold was used in the final Mendelian test, certain FS and GPO relationships could have been mistakenly identified as PO, particularly in the case of inbreeding. For example, the two accessions OH×F 247 and OH×F 512 appeared offspring of ‘Old Home’ × ‘Anjou’, and therefore different from all other rootstock accession of the same series. Interestingly, ‘Anjou’ is the parent of ‘Farmingdale’, the previously claimed parent of the OH×F series (Postman *et al.* 2013), thus it is possible that Anjou has a GPO, instead of PO, relationship with OH×F 247 and 512. Additionally, when documented year and origin of cultivars were unreliable or unavailable, it was not possible to determine the direction of the duos with certainty. Notes about ambiguous results have been appropriately reported in Table S3.

We found a high degree of inbreeding among the *P. communis* cultivars analyzed, with a small number of old pear cultivars as the main founders, and the same scenario was observed for Oriental *Pyrus* species. Despite the recent inbreeding, however, pear species are still highly heterozygous, likely because of their history of self-incompatibility (Wu *et al.* 2013; Chagné *et al.* 2014; Volk and Cornille 2019).

### A hierarchical population structure

The PC1 *vs.* PC2 plot for all the accessions (Figure 2a) showed a sample clustering very similar to what was observed in our previous work (Montanari *et al.* 2019) (where a smaller number of accessions was used), and mostly depicted the known groups of species identified by Challice and Westwood (1973). While the structure analysis reflected the results of the PCA, it also allowed a better representation of the genetic differentiation within some species groups (Figure 3). With both analysis, Occidental and Oriental accessions appeared genetically very different, reflecting their morphological diversity and their independent domestication events (Wu *et al.* 2018). These two major groups were themselves clusters of different subpopulations, which were depicted with the hierarchical approach.

The Occidental group included *P. communis sensu lato* and Groups 1 and 2, as described in Montanari *et al.* (2019) and in Table 2. Within the *P. communis* cluster, the two subspecies *pyraster* and *caucasica* formed two slightly distinct clusters closer to Group 1, although they appeared to largely overlap with several pure *P. communis* accessions (Figure 2b). In the structure analysis, these two subspecies formed their own subpopulations. While Challice and Westwood (1973) classified *P. nivalis* and *P. cordata* together with *P. communis* in the group “European species”, and *P. cossonii*, *P. gharbiana* and *P. mamorensis* together in the group “North African species” such classification was not confirmed, neither in the PCA nor from the structure analysis. In Montanari *et al.* (2019) all these species were assigned to Group 1 (Table 2), which formed a sparse cluster in between *P. communis* and Group 2 in the PC1 *vs.* PC2 plot (Figure 2b). In the structure analysis, the majority of *P. cordata* samples formed a subpopulation with *P. mamorensis*, while at the DAPC these two species could be clearly differentiated (Figure 4b). *P. nivalis* samples did not show a consistent organization, and only two samples each for *P. cossonii* and *P. gharbiana* were available and had complex hybrid structures, thus it was difficult to make any conclusion about these three species. Group 1 species *P. korshinskyi* also showed an unclear pattern. Group 2 formed a well-identifiable cluster in the PC1 *vs.* PC2 plot (Figure 2b), including the species *P. elaeagrifolia*, *P. sachokiana*, *P. salicifolia*, *P. spinosa* (syn. *P. amygdaliformis*) and *P. syriaca*. Challice and Westwood (1973) assigned all these species to the group “West Asian Species”, except for *P. sachokiana*. In the structure analysis, *P. elaeagrifolia* and *P. spinosa* stood out as two separate subpopulations, while *P. sachokiana* formed a subpopulation with *P. salicifolia* and *P. syriaca*. On the other hand, at the DAPC *P. syriaca* grouped with *P. elaeagrifolia* and not with *P. salicifolia* and *P. sachokiana* (Figure 4). Montanari *et al.* (2019) assigned to Group 2 also the species *P. glabra* and the hybrid *P*. ×*canescens* (Table 2). However, samples from *P. glabra* appeared admixed between Group 2 and *P. communis* subsp. *caucasica*, and the only one sample available for *P*. ×*canescens*and was likely a mis-classified *P. communis* accession. The species *P. regelii*, which was assigned to Group 2/“West Asian Species” (Table 2; Challice and Westwood (1973)), appeared to be quite distinct instead, forming its own cluster in between the Occidental and Oriental accessions (Figure 2a), as well as its own subpopulation among the Central and South Asian admixed group (Figure 2c and Figure 3).

The intergeneric hybrids that passed genotyping standards appeared to be admixed with a majority of Occidental ancestry. These included the ×*Pyronia* accession CIGC 9.001 (×*Pyronia veitchii*), which was reported as a *P. communis* × *Cydonia oblonga*, and the ×*Sorbopyrus* accession CIGC 28.001 (Pollwiller Pear), which was reported as *P. communis* × *Sorbus aria*. However, the fact that these accessions easily passed the genotyping thresholds applied for the *Pyrus* species might be an indication that they either have very small proportions of *Cydonia* and *Sorbus* genomes, or that they were mis-classified and actually are interspecific hybrids of two (or more) *Pyrus* species.

Within the Oriental major group, Groups 3 and 4 formed two clearly distinguishable clusters and subpopulations (Figure 2d and Figure 3). According to both Challice and Westwood (1973) and Montanari *et al.* (2019), Group 3 included the species *P. betulaefolia*, *P. calleryana*, *P. dimorphophylla*, *P. fauriei* and *P. koehnei*, and Group 4 the species *P. hondoensis*, *P. pashia*, *P. pyrifolia* and *P. ussuriensis* (Table 2). *P. betulaefolia* appeared distinct from the other Group 3 species, and was located farther away from the domesticated Group 4 accessions in the PC1 *vs.* PC2 plot (Figure 2d), indicating a possible more ancestral origin for this species. *P. calleryana* and *P. koehnei* were genetically similar, and so were *P. dimorphophylla* and *P. fauriei*. While the close grouping of *P. calleryana* and *P. koehnei* is not surprising, as they are also morphologically very similar, P. *dimorphophylla* and *P. fauriei* have distinct phenotypic characters and originate in different countries (Japan *vs.* Korea). The structure of Group 4 was a little more unclear, which could however be attributed to mis-classification of several accessions. *P. ussuriensis* was distinguishable from the other species, although there were several samples grouping with *P. pyrifolia* or appearing as hybrids of Group 4 species. Most of the *P. pyrifolia* accessions formed a subpopulation with the few samples of *P*. ×*sinkiangensis*, one of the major cultivated species in Asia, which was not reported by Challice and Westwood (1973). *P. hondoensis* samples were spread across the Group 4 cluster in the PC1 *vs.* PC2 plot (Figure 2d), and in the structure analysis they either grouped with *P. ussuriensis*, or appeared admixed with a majority of Group 4 ancestry. Group 4 also included *P*. ×*bretschneideri*, *P. pseudopashia* and *P. xerophila. P*. ×*bretschneideri* accessions appeared either admixed between *P. ussuriensis* and *P. pyrifolia*, or they were part of the *P. pyrifolia/ P*. ×*sinkiangensis* subpopulation. The other two Group 4 species showed inconsistent structural organization, casting doubts on their taxonomic classification.

Finally, a large number of true interspecific hybrids between Occidental and Oriental species could be confirmed or newly identified. The structure analysis highlighted a certain genetic similarity among hybrids of common geographical origin (Central and Southern Asia, Northern USA and Canada, Southern and Eastern USA, and Oregon, USA), probably as a result of breeding programs based on interspecific crosses or targeting adaptation to specific environmental conditions (University of Tennessee Agricultural Experiment *et al.* 1954; Westwood and Lombard 1977; Peteršon and Waples 1988; Bassil *et al.* 2008; Bell and Itai 2011). The assignment of some interspecific hybrids to their own species, such as *P*. ×*complexa*, *P*. ×*phaeocarpa* and *P*. ×*uyematsuana*, is arguable.

### Genetic diversity of the various species evaluated

Within the Occidental group, pure *P. communis* cultivars and accessions showed a wide diversity. Of all the 457 *P. communis* genotypes, the structure analysis separated only 39 of them into six distinguishable subpopulations (Communis A through E and Communis ‘Old Home’), which however could not be related to their geographic origin. On the other hand, the majority of the pure *P. communis* samples appeared to be admixed among these six subpopulations and were indicated as Admixed Communis in Figure 3. The *P. communis* accessions as a whole did not reveal a particular structure, as the attempt to identify subpopulations within them did not give any clear results (data not shown). The complexity of *P. communis* could not be resolved even with the DAPC, which returned ten different groups that, however, did not appear very diverse (Figure 4). There is confusion in the literature about subspecies *caucasica* and *pyraster*, which are considered by some as primary *Pyrus* species (Zheng *et al.* 2014; Wu *et al.* 2018), and by others as subspecies of *P. communis* (Challice and Westwood 1973; Asanidze *et al.* 2014). Our structure analysis suggested that they are genetically diverse from each other and from *P. communis*, enough to form their own subpopulations (Figure 3 and Figure 4), and they may therefore be considered as true species. *P. communis* subsps. *caucasica* and *pyraster* are believed to be the direct ancestors of the domesticated *P. communis* (Asanidze *et al.* 2014; Zheng *et al.* 2014), and the present study clearly showed that *pyraster* is more closely related to pure *P. communis* cultivars than *caucasica* (Figure 2b and Figure 4e). Challice and Westwood (1973) reported that several *P. communis* cultivars may also have originated from hybridization events between subspecies *caucasica* and *pyraster* with *P. nivalis*; however, it was not possible to confirm such hypothesis, since several *P. nivalis* accessions here evaluated appeared mis-classified, and the few remaining had an admixed ancestry between Group 2 species and *P. communis*, subsp. *caucasica* or subsp. *pyraster* (Table S4). It is worth noting, however, that the DAPC identified two separate clusters for *P. communis* subsp. *caucasica* (Figure 4), and one (P. communis caucasica B) was composed of accessions originally classified as P. nivalis.

Contradictory results were observed for the Group 1 species *P. cordata* and *P. mamorensis*, which appeared related to each other at the structure analysis (Figure 3), but very diverse at the DAPC (Figure 4b). Challice and Westwood (1973) believed that *P. cordata* had a central position in the evolution of *Pyrus*, being related to all Oriental and Occidental groups. However, such a unique connecting role could hardly be supported by the results of the present study. Analysis of more accessions from these two species will be necessary to better understand their relatedness and connection to other *Pyrus* species.

On the contrary to what Zheng *et al.* (2014) reported, we found P. *elaeagrifolia* was a well-defined species (Figure 3), although composed of two subgroups, one (CPYR 1482.001, 1483.001 and 1604.001) closer to *P. communis* and with a lower percentage of Oriental ancestry than the other one (Figure 2b). Also *P. spinosa* stood out as a subpopulation within the Occidental group, with its accessions being genetically very uniform, although they did not have any first-degree relationship with each other (Figure 1). *P. korshinskyi* accessions CPYR 2522.001 through 009 were re-assigned to *P. communis* subsp. *caucasica* by Volk *et al.* (2006). In our study, this classification was confirmed only for three of these accessions, while all other *P. korshinskyi* samples showed a more complex ancestry (although certainly involving subsp. *caucasica*), suggesting that it should not be considered as a true species. Challice and Westwood (1973) raised doubts about the classification of *P. glabra* as a true species as well, and its complex Group 2 and *P. communis* hybrid structure that resulted from the present study seems to confirm that hypothesis (Table S4). Finally, the species *P. salicifolia* and *P. sachokiana* were shown to be related, while *P. syriaca* might represent a connection between them and *P. elaeagrifolia* (Figure 2b, Figure 3 and Figure 4).

The classification of Group 3 was in accordance with what already reported by Challice and Westwood (1973), with *P. betulaefolia* the more clearly distinguishable species, *P. calleryana* related to P. *koehnei*, and *P. dimorphophylla* related to *P. fauriei* (which appeared as a true species, in disagreement with Wu *et al.* (2018)). However, it is possible that *P. betulaefolia* is the more ancient species within Group 3, as it is the most distant from the large-fruited Group 4 species (Figure 2d), which seems to be in disagreement with what was reported by Challice and Westwood (1973).

*P*. ×*bretschneideri* was long regarded as an interspecific hybrid (Challice and Westwood 1973; Zheng *et al.* 2014), and only recently had been reported as a true species (Wu *et al.* 2013, 2018). Results from the present study seems to reject the latter hypothesis, though, and in contrast support a *P. ussuriensis* × *P. pyrifolia* origin of *P*. ×*bretschneideri* (Table S4). Similarly, *P. hondoensis* also appeared to be a *P. ussuriensis* × *P. pyrifolia* hybrid. The conclusion that *P*. ×*sinkiangensis* is a hybrid between cultivated European and Asian pears (Wu *et al.* 2018) is not supported by this study, as only one out of eight analyzed samples had admixed ancestry. On the contrary, it appeared to be a Group 4 species, related to *P. pyrifolia* but distinct from it (Figure 2d and Figure 3). However, the number of *P*. ×*sinkiangensis* accessions was too low to make final conclusions about its origin and classification.

Very few samples were analyzed for *P. pashia*, *P. pseudopashia* and *P. xerophila*, and they all appeared either mis-classified or admixed, therefore preventing any further understanding of these species. All of the *P. xerophila* samples were seedlings from a single seedlot of uncertain provenance and tree phenotypes are consistent with that of *P. pyrifolia* hybrids.

The species *P. regelii* was probably the one that gave the most unexpected results. It was considered part of the “West Asian” (Group 2) species, although its morphology suggested it to be a divergent and more ancient species (Challice and Westwood 1973; Zheng *et al.* 2014). The structure analysis in the present study clearly showed *P. regelii* to have an admixed ancestry between Occidental and Oriental pears; however, it could be readily-separated from other hybrids and formed an unambiguous distinct subpopulation (Figure 2a, c and Figure 3). This is somehow in contrast with that reported by Wu *et al.* (2018), who suggested that the highly admixed ancestry of *P. regelii* was an indication of its re-classification as an “interspecies”, rather than a true species. In view of the structure analysis, it is more likely that *P. regelii* is a true species that resulted from hybridization of ancestral Oriental and Occidental pears and remained isolated, or a connecting link between the two major groups of species.

## Conclusions

This is the first study that genetically characterized the entire *Pyrus* germplasm collection held at the NCGR, one of the largest *Pyrus* repository in the world. The in depth genotyping performed with the Axiom Pear 70 K Genotyping Array (Montanari *et al.* 2019) allowed the identification of several duplicated samples in the collection. Those that have been flagged as possible sampling errors will be verified by comparison of the morphology of the original trees at the NCGR collection and/or by SSR fingerprinting, as in Montanari *et al.* (2019). This information will be particularly useful for the optimization of the conservation strategy at the repository. Additionally, by analyzing a large number of samples, this study was able to reconstruct the parentage (or partial parentage) of 637 accessions, giving insights into the level of inbreeding in cultivated pear. Pear breeders across the world will be able to use this extended pedigree to make more informed decisions in their crossing schemes, while maximizing efforts to maintain diversity within their programs. The population structure analysis, made possible by the high quality of the SNPs included in the Axiom Pear 70 K Genotyping Array, enabled the re-classification of a large number of accessions and improved our understanding of the genetic diversity of *Pyrus* species. Further analysis of this dataset in conjunction with morphological and phenological data will be performed to better evaluate the genetic diversity of the different *Pyrus* species. Phylogeneticists and taxonomists can build on the information reported here to better elucidate the evolution and domestication of pear.

### Additional Information

We found that the large and complex pedigree network built in this work was better represented with the software Helium (Shaw *et al.* 2014), which allows interactive visualization. The software is downloadable for free at https://github.com/cardinalb/helium-docs/wiki and the input files are given in Files S1-S5.
